# Dietary isotopes of Madagascar's extinct megafauna reveal Holocene browsing and grazing guilds

**DOI:** 10.1098/rsbl.2022.0094

**Published:** 2022-04-13

**Authors:** James P. Hansford, Samuel T. Turvey

**Affiliations:** ^1^ Institute of Zoology, Zoological Society of London, Regent's Park, London NW1 4RY, UK; ^2^ Department of Biological Sciences, Northern Illinois University, DeKalb, IL 60115, USA; ^3^ School of Ocean and Earth Science, National Oceanography Centre, University of Southampton, Southampton, UK

**Keywords:** elephant bird, hippopotamus, isotope ecology, megafauna, quaternary extinction

## Abstract

Megafauna play a disproportionate role in developing and maintaining their biomes, by regulating plant dispersal, community structure and nutrient cycling. Understanding the ecological roles of extinct megafaunal communities, for example through dietary reconstruction using isotope analysis, is necessary to determine pre-human states and set evidence-based restoration goals. We use *δ*^13^C and *δ*^15^N isotopic analyses to reconstruct Holocene feeding guilds in Madagascar's extinct megaherbivores, which included elephant birds, hippopotami and giant tortoises that occurred across multiple habitats and elevations. We compare isotopic data from seven taxa and two elephant bird eggshell morphotypes against contemporary regional floral baselines to infer dietary subsistence strategies. Most taxa show high consumption of C_3_ and/or CAM plants, providing evidence of widespread browsing ecology. However, *Aepyornis hildebrandti*, an elephant bird restricted to the central highlands region, has isotope values with much higher *δ*^13^C values than other taxa. This species is interpreted as having obtained up to 48% of its diet from C_4_ grasses. These findings provide new evidence for distinct browsing and grazing guilds in Madagascar's Holocene megaherbivore fauna, with implications for past regional distribution of ecosystems dominated by endemic C_4_ grasses.

## 1. Introduction

Late Quaternary and older terrestrial ecosystems were typically dominated by megaherbivores, which shaped their environments through top–down interactions with plant communities and vegetation structure [1,2]. Megaherbivores impact diversity and structure of ecosystems by suppressing plant growth through physical disturbance and herbivory, influencing nutrient cycling within and between landscapes, and dispersing plant propagules [3,4]. However, megafauna have been disproportionately vulnerable to human-caused extinction, and many ecosystems now lack these keystone species [5]. Reconstructing the ecology of now-extinct megaherbivore guilds is essential to identify disrupted ecological processes and guide environmental management and restoration [1,6].

Late Quaternary Madagascar supported a diverse megafauna, including elephant birds, hippopotami and giant tortoises. Madagascar's megaherbivores became extinct in the late Holocene during a period of intensive anthropogenic forest clearance and conversion to open habitats around 1100–1000 BP [7–9]. Today 80% of Madagascar is covered by grassland [10,11], but the pre-disturbance distribution and extent of native grasslands remain uncertain [12,13]. Madagascar contains native grass lineages dating from the Miocene, and 40% of its grass species are unique; it contains among the world's highest grass diversity and endemism, with particularly diverse assemblages in the island's central highlands ecogeographical region [10,11]. However, there is limited evidence for regional existence of a late Quaternary vertebrate grazing guild, suggesting that endemic grasses may have been limited to small, low-density clearings [13–16].

Most trees, shrubs and herbs use a C_3_ (Calvin) metabolic pathway for carbon fixation during photosynthesis, whereas most tropical grasses use a C_4_ (Hatch-Slack) pathway [17]. Stable carbon isotope (*δ*^13^C) values in bones of animals that consumed these plants can indicate likely former presence of forests or grasslands, and isotope analysis is widely used for late Quaternary palaeoecological reconstruction [14]. Animals with pure C_3_ diets have *δ*^13^C values below −21.5‰ and pure C_4_ diets above −9‰. Reported *δ*^13^C values for Madagascar megaherbivores are interpreted as indicating forest environments [18–20]. However, some Madagascar megaherbivore subfossil sites are interpreted as open grassland biomes [21,22]. Using *δ*^13^C values to infer open-habitat grasses as dietary resources can be confused by plants using crassulacean acid metabolism photosynthesis (CAM plants); for example, the succulent plant *Kalanchoë* exhibits flexible CAM patterns across Madagascar, with *δ*^13^C values similar to C_3_ plants in humid environments and to C_4_ plants in dry environments [23]. The CAM-specialist extinct lemur *Hadropithecus* shows *δ*^13^C values of −24.2‰ in the mesic central highlands, and −9.6‰ in the arid southwest [14]. However, wetland C_4_ plants (rushes, sedges) also occur in Madagascar, so comparison of *δ*^15^N values between co-occurring taxa can also be included in dietary assessments using *δ*^13^C data; plants in arid environments have higher *δ*^15^N values [24], enabling differentiation between wetland and dryland C_4_ plants.

Research into Madagascar's extinct vertebrate ecology has mainly focused on giant lemurs, with studies of megaherbivores hindered by poorly resolved taxonomy [25]. However, recent taxonomic reassessments have clarified species diversity in elephant birds [26] and hippopotami [27], enabling the investigation of species-specific niches and landscape ecology. Here we investigate new and published dietary isotope data for all Madagascar hippopotamus and elephant bird species and for the regionally extinct giant tortoise *Aldabrachelys* across three distinct ecogeographical zones, to determine megaherbivore dietary niches and presence of natural open grassland habitats (e.g. savannahs, open wooded habitats) in Madagascar's late Quaternary ecosystems [25].

## 2. Material and methods

**Figure 1.**
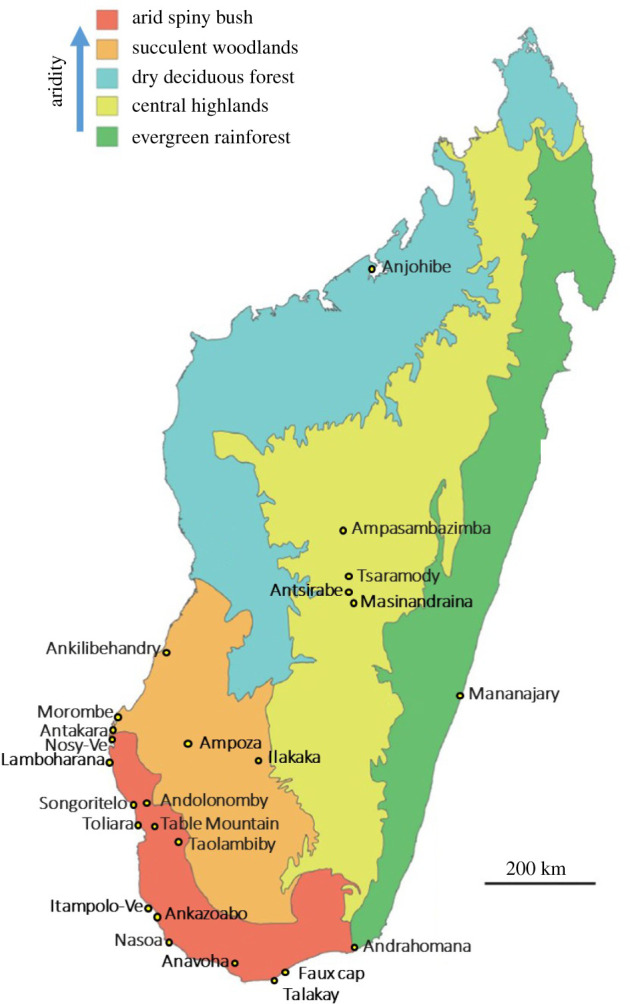
Madagascar ecoregions, showing localities for specimens in this study. Adapted from [29].

We assembled a database of 203 *δ*^13^C and 118 *δ*^15^N values for late Quaternary skeletal elements of all recognized Madagascar hippopotami (*Hippopotamus lemerlei*, *n* = 15; *H. madagascariensis*, *n* = 6), elephant birds (*Aepyornis hildebrandti*, *n* = 8; *A. maximus*, *n* = 3; *Mullerornis modestus*, *n* = 9; *Vorombe titan*, *n* = 11) and *Aldabrachelys* sp. (*n* = 19), and for both elephant bird eggshell morphotypes (‘thin eggshell’ representing *M. modestus*, *n* = 9; ‘thick eggshell’ representing *Aepyornis* or *Vorombe*, *n* = 93; [28]) (table 1)*.* We include 243 published values (160 *δ*^13^C, 83 *δ*^15^N), and previously unreported data for 42 specimens from accelerator mass spectrometry (AMS) dating of bone collagen [9] performed at the Oxford Radiocarbon Accelerator Unit (ORAU). In total, 86 samples have associated radiocarbon dates, with just one predating the Holocene (NIUTSM 01539: 14 580 ± 460 BP). Collection localities cover three Madagascan ecoregions: southern arid spiny bush (*n* = 147, 14 localities), western succulent woodland (*n* = 25, six localities) and the central highlands (*n* = 21, four localities) (figure 1; electronic supplementary material, table S1). Ecoregions varied in megaherbivore composition; although *Mullerornis* occurred in highland and lower-elevation regions, *A. hildebrandti* and *H. madagascariensis* were largely/completely restricted to the central highlands, and most other species only occurred in southern and/or western ecoregions [25,30] (table 1). *Aldabrachelys* data are only available for the arid spiny bush, although specimens are also recorded from the central highlands [28] (table 1).

**Table 1.** Mean isotope values and dietary proportion estimates for Madagascar's megaherbivores, inclusive of bone/eggshell correction and Suess correction.

taxon	no. specimens	*δ*^13^C (mean)	s.d.	est. diet proportion (mean) C_3_ plants	est. diet proportion (mean) CAM plants	s.e.
1. Arid spiny bush						
*Aepyornis maximus*	2	−28.42	0.71	0.93	0.07	0.04
*Mullerornis modestus* (bone)	7	−26.44	0.81	0.79	0.21	0.02
*Mullerornis modestus* (eggshell)	9	−25.58	0.48	0.73	0.27	0.01
thick eggshell	93	−26.35	0.92	0.79	0.21	0.01
*Hippopotamus lemerlei*	10	−21.87	2.49	0.48	0.52	0.06
*Hippopotamus* sp.	18	−22.99	2.33	0.55	0.45	0.04
*Aldabrachelys* sp.	18	−25.18	2.75	0.71	0.29	0.05
2. Succulent woodland						
*Aepyornis maximus*	1	−28.02	0.71^a^	0.9	0.1	0.05
*Vorombe titan*	11	−29.26	0.72	0.99	0.01	0.02
*Hippopotamus lemerlei*	4	−28.13	0.73	0.91	0.09	0.03
*Hippopotamus madagascariensis*	3	−20.17	1.56	0.36	0.64	0.06
*Hippopotamus* sp.	15	−26.07	3.71	0.77	0.23	0.07
*Aldabrachelys* sp.	1	−33.52	2.75^a^	1	0	0.19

taxon	no. specimens	*δ*^13^C (mean)	s.d.	est. diet proportion (mean) C_3_ plants	est. diet proportion (mean) CAM plants	s.e.
3. Central highlands						
*Aepyornis hildebrandti*	8	−21.12	1.42	0.52	0.48	0.03
*Mullerornis modestus* (bone)	1	−28.22	0.81^a^	0.98	0.02	0.06
*Hippopotamus madagascariensis*	3	−28.27	3.25	0.99	0.01	0.12
*Hippopotamus* sp.	13	−27.83	4.29	0.96	0.04	0.08

^a^s.d. not available and arid spiny bush value used.

We assessed dietary sources using mixing models in ISSOERROR v. 1.04 [31] to investigate dietary niche differentiation between taxa within ecoregions. We calculated proportionate consumption of C_3_ versus CAM plants in the more arid southern and western ecoregions, and C_3_ versus C_4_ plants in the comparatively wet central highlands, which do not support significant CAM plant biomass and contain a regionally restricted endemic C_4_ plant community [10,11,14,15]. We used *δ*^13^C isotope values for discrimination model end-members from ref. [32]: C_3_ plants, arid spiny bush and succulent woodland (Beza Mahafaly), −29.4‰ (*σ*: 2.4, *n* = 240); central highlands (Tsinjoarivo): −28.5‰ (*σ*: 1.8, *n* = 49); CAM plants, arid spiny bush and succulent woodland (Beza Mahafaly): −15‰ (*σ*: 1.2, *n* = 67). C_4_ grass end-member values used the global mean value of −13.1‰ [33], with substituted *σ* and sample size from central highlands C_3_ plants. *δ*^13^C values used in fractionation were corrected to account for *δ*^13^C enrichment in bone (+5‰) [34] and eggshell (+2‰) collagen [35], and by +1.22‰ to account for *δ*^13^C shifts in atmospheric CO_2_ (Suess effect; [36]).

## 3. Results

**Figure 2.**
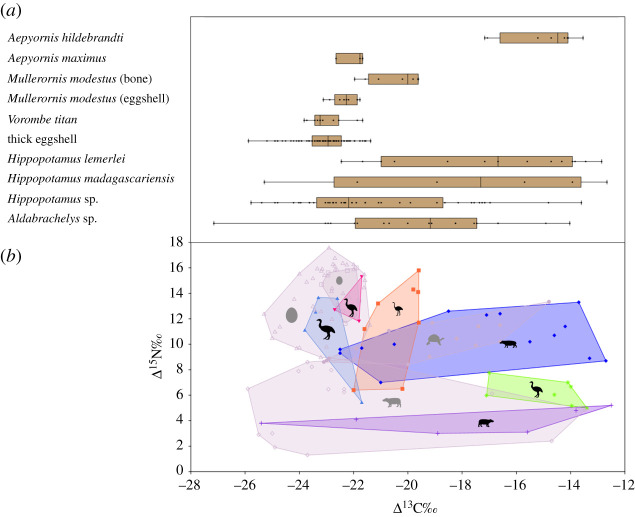
Observed isotope ‰ for Madagascar's megaherbivores: (*a*) *δ*^13^C values. (*b*) *δ*^13^C and *δ*^15^N values. Key: green star, *Aepyornis hildebrandti*; pink triangle, *A. maximus*; orange square, *Mullerornis modestus* (bone); blue triangle: *Vorombe titan*; open square: thin eggshell (*M. modestus*); open triangle: thick eggshell (*Aepyornis* or *Vorombe*); blue diamond: *Hippopotamus lemerlei*; purple cross: *H. madagascariensis*; open diamond: *Hippopotamus* sp.; filled circle: *Aldabrachelys* sp.

In arid spiny bush, elephant birds and giant tortoises show low *δ*^13^C values (species means: −25.18 to −28.42‰), with dietary fractionation indicating these taxa all consumed mainly C_3_ plants and only limited amounts of CAM plants (mean estimated proportions of CAM consumption: 0.07–0.27). *M. modestu*s (bone and thin eggshell) shows highest estimated CAM consumption (sample means: 0.21–0.27). Hippopotami (*H. lemerlei* and samples unidentified to species) show higher *δ*^13^C values (sample means: −21.87 to −22.99‰) and correspondingly much higher estimated proportions of CAM consumption, with C_3_ and CAM plants both comprising about half of their diet (mean estimated proportions, C_3_: 0.48–0.55, CAM: 0.45–0.52) (table 1 and figure 2; electronic supplementary material, file S1).

In succulent woodland, elephant birds (*A. maximus, V. titan*), giant tortoises and *H. lemerlei* show low *δ*^13^C values (species means: −26.46 to −33.52‰) and very low estimated proportions of CAM consumption (0.01–0.09). By contrast, *H. madagascariensis* shows high *δ*^13^C values (species mean: −20.16‰) and much higher mean estimated CAM consumption (0.64).

In the central highlands, *M. modestus* and hippopotami (*H. madagascariensis* and samples unidentified to species) show high *δ*^13^C values (sample means: −28.22 to −28.83‰), and are estimated to have consumed almost entirely C_3_ plants and minimal C_4_ grasses (mean estimated proportions of C_4_ consumption: 0.02–0.04). Conversely, *A. hildebrandti* shows high *δ*^13^C values (sample mean: −21.12‰) and much higher mean estimated C_4_ consumption (0.48).

For hippopotami, *H. madagascariensis* had *δ*^15^N values of 3.0–5.2‰, and *H. lemerlei* of 7.0–13.3‰. The lowest hippopotamus *δ*^15^N value was from Antsirabe, central highlands (1.3‰) and the highest was from Beloha, arid spiny bush (13.3‰). For elephant birds, *A. hildebrandti* had *δ*^15^N values of 5.0–7.8‰, *A. maximus* of 11.8–15.3‰, *M. modestus* of 6.4–16.0‰, *V. titan* of 5.5–13.7‰ and ‘thick eggshell’ of 8.7−17.6‰. *Aldrabrachely*s in arid spiny bush had values of 8.4–13.3‰. Across ecoregions, non-overlapping *δ*^15^N ranges were observed for single measurements in *A. maximus* (dry deciduous forest, 11.8‰; arid spiny bush, 12.7–15.3‰) and *M. modestus* (central highlands, 6.4‰; arid spiny bush, 6.5–15.8‰).

## 4. Discussion

We present the first species-level dietary niche reconstruction for Madagascar's megaherbivores, revealing a range of *δ*^13^C and *δ*^15^N values and trophic ecologies across taxa and ecoregions. Our findings indicate the former existence of multiple herbivore guilds across Madagascar. These data support previous identification of a widespread browsing guild and provide the first direct evidence for a megaherbivore grazing guild in Madagascar's central highlands.

*δ*^13^C values in hippopotamus samples indicate broad trophic niches for both species, suggesting both browsing and grazing behaviours. This contrasts with the mainland African hippopotamus (*H. amphibius*), which is predominantly a terrestrial grazer [37]. Madagascar hippopotami were trophically closer to Africa's extant pygmy hippopotamus (*Choeropsis liberiensis*), which is comparable in size to Madagascar's extinct species, and browses on forest plants [38]. Interestingly, *δ*^15^N ratios indicate more aquatic feeding in *H. madagascariensis* than *H. lemerlei*. Aquatic habitats were available for *H. madagascariensis* in the central highlands [25]. This result contrasts with aquatic adaptations inferred from cranial morphology in *H. lemerlei* [39], but is consistent with behavioural ecology (emergence onto land for feeding) in the otherwise aquatic *H. amphibius*, suggesting a similar lifestyle for *H. lemerlei*. *Aldabrachelys* isotopes from arid spiny bush show a comparable *δ*^13^C/*δ*^15^N signal to *H. lemerlei*, indicating a similar browsing niche in this ecoregion.

CAM plants comprised a substantial proportion of the diets of one or both hippopotamus species in arid spiny bush and succulent woodland, but *δ*^13^C values are lower in the central highlands, suggesting higher reliance upon C_3_ plants. CAM plants are relatively scarce in this region; however, they occur across numerous biomes and elevations in Madagascar, with a range of *δ*^13^C values (e.g. *Kalanchoë*, −11.4 to −27.3‰) [23]. CAM plant CO_2_ is fixed by the C_3_ pathway in humid environments such as the central highlands, producing *δ*^13^C values below −22‰ [23] and thus consistent with CAM consumption in this region as well. Hippopotami therefore probably consumed C_3_ and CAM plants across Madagascar, matching the varying *δ*^13^C pattern in the CAM-specialist *Hadropithecus* across different ecoregions [14].

Elephant bird *δ*^13^C values from arid spiny bush and succulent woodland fall outside the range for C_4_ consumption (open-habitat dryland grasses or wetland sedges and rushes). In arid spiny bush, *δ*^13^C values indicate that all elephant birds had predominantly C_3_ diets, with some CAM consumption by *M. modestus*; higher *δ*^15^N values compared to sympatric hippopotami indicate that these plants were less likely to be from wetlands. Differences between sympatric elephant birds may indicate further species-specific dietary differences; for example, higher *δ*^15^N values (e.g. in *A. maximu*s) are associated with frugivory or omnivory (including invertebrate or small vertebrate consumption) [32], which comprise extant ratite dietary strategies [40]. Eggshell and bone values also differ in *M. modestus*, possibly indicating seasonal reliance upon dietary resources during oogenesis, or that eggshell and bone fractionation rates may need separate assessment.

Our most striking result is that *δ*^13^C data for *A. hildebrandti* provide the first evidence for grazing ecology in elephant birds. Although unique within Madagascar's ratites, grazing is also the primary dietary strategy in greater rhea (*Rhea americana*) [41], and other large flightless birds (e.g. geese) also have important regulatory effects on island grasslands [42]. Our results thus identify *A. hildebrandti* as a likely top–down regulator of native grassland ecosystems in the central highlands [13,15,16]. *δ*^13^C values for this species indicate a mixed diet containing large quantities of C_4_ plants (c. 48%), whereas co-occurring hippopotami consumed only tiny amounts of C_4_ plants (1–4%). Although *A. hildebrandti* had higher mean *δ*^15^N values compared to sympatric hippopotami, this disparity is much lower than between species in other ecoregions. Indeed, lower *δ*^13^C values in CAM plants within mesic conditions [23] suggest that *A. hildebrandti* might not have consumed any forest plants and was exclusively an open-habitat forager, consuming a mixture of C_4_ and CAM plants. High variability in CAM plant *δ*^13^C values complicates interpretation of results, but the likelihood of *A. hildebrandti* exhibiting grazing behaviour is supported by the non-matching regional *δ*^13^C signature of the CAM specialist *Hadropithecus* (mean: −24.2 *δ*^13^C‰) [14]. This hypothesis is consistent with the small olfactory bulb in skulls assigned to *A. hildebrandti*, comparable to the neuroanatomy of extant open-habitat palaeognaths [43]. However, the taxonomic identity of these crania is uncertain; they are not associated with diagnostic postcrania or locality data, and two separate skull morphotypes have been referred to *A. hildebrandti* [44–46].

*δ*^13^C data from skeletal collagen provide a comprehensive new understanding of Madagascar megaherbivore dietary ecology. Most available subfossils originate from southern Madagascar, and further research should investigate data across wider areas. For example, giant tortoises from the central highlands remain isotopically unstudied; these animals might also have been grazers, but their shell shape (associated with biomechanical advantage for grazing or browsing in extant species [47]) is poorly understood, making ecological inference difficult. However, whereas most modern-day open habitats on Madagascar are anthropogenic in origin, our results provide important evidence for former existence of native ecosystems dominated by endemic C_4_ grasses. It is clear that Madagascar supported multiple megaherbivore trophic guilds with differing relationships to native vegetation, which must have played important roles in regulating diverse natural landscapes. Madagascar's ecosystems are now highly degraded, and protection and sustainable management of landscapes and ecosystem services represents a global priority for biodiversity conservation and human well-being [48,49]. Hypotheses of what constitutes a ‘natural’ Madagascar ecosystem must therefore consider the ecologies and regulatory roles of the island's now-extinct megafauna, to support evidence-based restoration of this ecologically complex island.

## Data Availability

All data are available in electronic supplementary material, file S1 [50].
